# Risk Factors for Severe Pyometra Uteri in Elderly Women: A Case-Control Study at a Tertiary Care Hospital in Japan

**DOI:** 10.7759/cureus.105401

**Published:** 2026-03-17

**Authors:** Hiroshi Mori, Ayako Imai

**Affiliations:** 1 Department of Obstetrics and Gynecology, Kenwakai Otemachi Hospital, Kitakyusyu, JPN

**Keywords:** acute care medicine, elderly women, female urology and uro oncology, geriatric sepsis, japanese geriatrics, pyometra uteri, uterine rupture

## Abstract

Background

Pyometra uteri (PU) is an uncommon gynecologic condition characterized by purulent accumulation within the uterine cavity. Although it is typically managed conservatively with drainage and antibiotics, severe cases - particularly those complicated by uterine rupture (UR) - are associated with substantial morbidity and high mortality. Evidence regarding factors associated with severe PU remains limited, especially in aging populations. This study, therefore, explored clinical and microbiological factors potentially associated with severe PU in elderly women.

Methods

This retrospective case-control study included 120 patients diagnosed with PU at a tertiary-care hospital in Japan between January 2014 and September 2024. Severe cases were defined as those requiring intensive care unit (ICU) or high care unit (HCU) admission. Clinical characteristics, microbiological findings, and outcomes were compared between severe (n=10) and non-severe (n=110) cases. Because of the limited number of severe cases, multivariable logistic regression was not feasible; therefore, univariate logistic regression analyses were performed, with additional age- and BMI-adjusted stratified analyses using the Mantel-Haenszel method. Receiver operating characteristic (ROC) curve analysis was used to explore the predictive performance of abscess size.

Results

The median age of the study population was 84 years. Ten patients (8.3%) were classified as severe; and all had UR. The in-hospital mortality rate among severe cases was 50%. Several clinical and microbiological variables were statistically associated with severe disease in univariate analyses, including fever, abdominal pain, presence of coexisting adenomyosis, detection of extended-spectrum β-lactamase (ESBL)-producing *Escherichia coli* in vaginal pus, positive urine culture, and increasing abscess size. ROC analysis suggested a possible abscess size threshold of 77 mm (area under the curve or AUC=0.77), with 60% sensitivity and 91% specificity for identifying severe cases. However, this cutoff was derived from the present dataset and requires external validation.

Conclusions

Systemic symptoms, larger abscess size, certain gynecologic comorbidities, and microbiological findings may be associated with severe PU. Given the small number of severe cases and the exploratory nature of the analyses, these findings should be interpreted cautiously and considered hypothesis-generating. Larger prospective studies are needed to validate these observations.

## Introduction

Pyometra uteri (PU) is an uncommon gynecologic condition characterized by the accumulation of purulent material within the uterine cavity due to impaired drainage of uterine secretions. The condition most commonly occurs in postmenopausal women and is frequently associated with cervical obstruction caused by malignancy, cervical stenosis related to aging, or chronic inflammatory conditions [1].

Other reported contributing factors include puerperal infections, radiation-induced cervicitis, long-term intrauterine device (IUD) use, congenital uterine anomalies, and tubo-ovarian abscesses [2-6]. Clinically, PU may present with purulent vaginal discharge, postmenopausal bleeding, lower abdominal pain, or uterine enlargement. However, a substantial proportion of patients may remain asymptomatic, particularly elderly individuals with limited ability to communicate symptoms or reduced inflammatory responses [7].

Although PU is generally considered a benign and manageable condition, serious complications can occur. The most severe complication is uterine rupture (UR), which can lead to peritonitis, septic shock, and death. Previous reports have suggested that mortality associated with ruptured PU may exceed 20%, particularly in elderly patients with multiple comorbidities [8].

Despite its potential severity, the current literature on PU largely consists of individual case reports and small case series. Consequently, clinical factors associated with severe disease remain poorly understood, and evidence to guide early identification of high-risk patients is limited. This issue may become increasingly relevant in aging societies such as Japan, where the proportion of elderly women continues to increase [9].

Therefore, the primary objective of the present study was to explore clinical factors associated with severe PU by comparing severe and non-severe cases diagnosed at a tertiary-care hospital. In this study, severe PU was defined as cases requiring intensive care unit (ICU) or high care unit (HCU) admission; notably, all severe cases in the present cohort were complicated by UR. In addition to this primary objective, the secondary objectives were to describe the microbiological characteristics of PU, to examine potential associations between microbiological findings and disease severity, and to summarize the clinical management of severe cases, including surgical approaches and outcomes. Because severe cases were rare in this cohort, the analyses were designed as an exploratory investigation intended to generate hypotheses for future research rather than to establish definitive risk factors.

## Materials and methods

Study design and population

This retrospective observational case-control study was conducted at Kenwakai Otemachi Hospital, a tertiary-care hospital in Japan. The study included consecutive patients diagnosed with PU between January 2014 and September 2024. PU was diagnosed when both of the following criteria were met: (1) clinical evidence of purulent cervical discharge or drainage, and (2) radiologic evidence of intrauterine fluid accumulation compatible with pus on ultrasonography or computed tomography (CT). Patients were excluded if intrauterine fluid accumulation was attributable to noninfectious conditions such as hematometra or hydrometra, if radiologic confirmation was unavailable, or if essential clinical information was missing from the medical records. For the purposes of this study, severe PU was operationally defined as PU requiring admission to the ICU or HCU for hemodynamic instability, septic shock, or the need for intensive monitoring and organ support. In the present cohort, all severe cases were complicated by UR. Therefore, the analysis primarily reflects factors associated with severe PU accompanied by UR rather than severe PU in general. Given the rarity of severe cases, patients with severe PU were compared with patients with non-severe PU treated during the same study period. This study was approved by the Kenwakai Otemachi Hospital Ethics Committee (Approval No. 24003).

Data collection

Clinical data were retrospectively extracted from electronic medical records. Collected variables included: demographic characteristics (age, parity, BMI), baseline functional status assessed using the Eastern Cooperative Oncology Group performance status (ECOG PS) scale, residential status before disease onset, presenting symptoms, gynecologic and general comorbidities, microbiological culture results (vaginal pus, urine, and blood cultures), treatment details, and clinical outcomes.

Abscess size was defined as the maximum diameter of the intrauterine fluid collection measured on imaging. Measurements were obtained from either transvaginal or transabdominal ultrasonography or computed tomography (CT), depending on the imaging modality performed in routine clinical care. When multiple imaging studies were available, the largest recorded measurement before therapeutic intervention was used for analysis. Because this was a retrospective study conducted in routine clinical practice, imaging protocols were not fully standardized. Measurements were based on radiologic reports and physician documentation in the medical records.

Microbiological assessment

Microbiological samples were obtained as part of routine clinical care. Vaginal pus samples were collected during cervical drainage procedures when possible. Urine and blood cultures were performed when clinically indicated. Bacterial identification and antimicrobial susceptibility testing were conducted in the hospital microbiology laboratory according to standard laboratory procedures. Extended-spectrum β-lactamase (ESBL) production was determined using routine susceptibility testing methods used by the laboratory during the study period.

Sample size considerations

The sample size calculation was based on the expected proportion of exposure among controls and an estimated odds ratio (OR) derived from previously published literature on uterine rupture associated with PU. Assuming a control exposure rate of approximately 18.5% [10] and an OR of 8.0 [11], a case-control ratio of 1:11 was selected to improve statistical efficiency given the rarity of severe cases. Under these assumptions, a total sample size of approximately 120 patients (10 cases and 110 controls) was estimated to provide 80% statistical power at a two-sided significance level of 0.05. The calculation was performed using OpenEpi software (Dean AG, Sullivan KM, Soe MM. OpenEpi: Open Source Epidemiologic Statistics for Public Health, Version 3.01. www.OpenEpi.com, updated 2013/04/06). Because the present study included all eligible PU cases treated during the study period, the final sample size corresponded to the total number of available patients.

Statistical analysis

Continuous variables were summarized as medians with ranges and compared using the Mann-Whitney U test. Categorical variables were expressed as counts and percentages and compared using Fisher’s exact test. Associations between clinical variables and severe PU were initially evaluated using univariate logistic regression, and crude ORs with 95% confidence intervals (CIs) were calculated. Because the number of severe cases was limited, a full multivariable logistic regression model could not be reliably performed. Therefore, logistic regression analyses adjusted for age and BMI were conducted to account for potential confounding. Receiver operating characteristic (ROC) curve analysis was used to explore the ability of abscess size to discriminate between severe and non-severe cases. The optimal cutoff value was estimated using the Youden index. Because this cutoff was derived from the study dataset, it should be interpreted as exploratory and requires external validation. A two-sided p-value <0.05 was considered statistically significant. All statistical analyses were performed using BellCurve for Excel (version 4.06; Social Survey Research Information Co., Ltd., Tokyo, Japan).

## Results

Patient characteristics

A total of 120 patients diagnosed with PU were included in the study. Among them, 10 patients (8.3%) required ICU admission and were classified as severe cases; the remaining 110 were classified as non-severe. Table 1 summarizes the patients' backgrounds and the results of the comparison between the severe and non-severe groups.

**Table 1:** Characteristics of the study population (patients with PU) Data are presented as n (%) or median (minimum-maximum). The p-value is a comparison of severe and non-severe cases; *p< 0.05, **p< 0.01. ADL, activities of daily living; BMI, body mass index; ESBL, extended-spectrum β-lactamase; IUD, intra-uterine device; MRSA, methicillin-resistant *Staphylococcus aureus*; PS, performance status; PU, pyometra uteri

	All cases (n=120)	Severe cases (n=10)	Non-severe cases (n=110)	p-value
Age, years	84 (50-105)	78.5 (50-92)	84 (63-105)	0.09
Parity	2 (0-5)	2 (0-3)	2 (0-5)	0.92
BMI (kg/m^2^)	17.9 (11-27.9)	19.4 (15.7-21)	17.9 (11-27.9)	0.39
Abscess size (longest axis, mm)	33 (10-111)	77.5 (22-111)	31.5 (10-110)	0.004**
					Pre-onset ADL				
PS 4	55 (46%)	3 (30%)	52 (47%)	0.34
PS 3-4	100 (83%)	6 (60%)	94 (85%)	0.061
PS 3	45 (38%)	3 (30%)	42 (38%)	0.74
PS 2	13 (11%)	1 (10%)	12 (11%)	1
PS 0-2	20 (17%)	4 (40%)	16 (15%)	0.061
PS 1	3 (2.5%)	1 (10%)	2 (1.8%)	0.23
PS 0-1	7 (5.8%)	3 (30%)	4 (3.6%)	0.012*
PS 0	4 (3.3%)	2 (20%)	2 (1.8%)	0.035*
Residential settings				
nursing home	79 (66%)	6 (60%)	73 (66%)	0.73
long-term care sanatorium	12 (10%)	1 (10%)	11 (10%)	1
home (independent living)	19 (16%)	3 (30%)	16 (15%)	0.19
rehabilitation hospital	10 (8.3%)	0	10 (9%)	1
Symptoms				
abnormal vaginal discharge	95 (79%)	6 (60%)	89 (81%)	0.21
atypical genital bleeding	27 (23%)	2 (20%)	25 (23%)	1
fever (>38.0℃）	26 (22%)	9 (90%)	17 (15%)	<0.001
none	9 (7.5%)	0	9 (8.1%)	1
abdominal pain	6 (5%)	4 (40%)	2 (1.8%)	<0.001
General comorbidities				
hypertension	71 (59%)	7 (70%)	64 (58%)	0.52
diabetes mellitus	21 (18%)	0	21 (19%)	0.21
hyperlipidemia	13 (11%)	2 (20%)	11 (10%)	0.29
cerebral vascular diseases	47 (39%)	1 (10%)	46 (42%)	0.086
chronic heart diseases	33 (28%)	4 (40%)	29 (26%)	0.46
chronic liver diseases	7 (5.8%)	0	7 (6.4%)	1
chronic kidney diseases	10 (8.3%)	1 (10%)	9 (8.2%)	0.59
maintenance hemodialysis	4 (3.3%)	0	4 (3.6%)	1
osteoporosis	36 (30%)	3 (30%)	33 (30%)	1
dementia	104 (87%)	6 (60%)	98 (89%)	0.028*
Gynecological comorbidities				
uterine cancer	3 (2.5%)	1 (10%)	2 (1.8%)	0.23
pelvic organ prolapse	2 (1.7%)	0	2 (1.8%)	1
IUD left in the uterus	3 (2.5%)	0	3 (2.5%)	1
adenomyosis	3 (2.5%)	2 (20%)	1 (0.9%)	0.018*
uterine myoma	13 (11%)	2 (20%)	11 (10%)	0.29
Culture of vaginal pus				
Escherichia coli	24 (20%)	3 (30%)	21 (19%)	0.42
Escherichia coli (ESBL)	12 (10%)	4 (40%)	8 (7.3%)	0.009**
Klebsiella pneumoniae	4 (3.3%)	1 (10%)	3 (2.7%)	0.2972
Klebsiella pneumoniae (ESBL)	1 (0.8%)	0	1 (0.9%)	1
Proteus mirabilis (ESBL)	1 (0.8%)	0	1 (0.9%)	1
Enterococcus faecalis	2 (1.7%)	1 (10%)	1 (0.9%)	0.16
Staphylococcus spp.	3 (2.4%)	0	3 (2.7%)	1
MRSA	1 (0.8%)	0	1 (0.9%)	1
Streptococcus spp.	4 (3.3%)	0	4 (3.6%)	1
Corynebacterium spp.	6 (5%)	0	6 (5.5%)	1
Prevotella spp.	2 (1.7%)	0	2 (1.8%)	1
Bacteroides spp.	5 (4.2%)	1 (10%)	4 (3.6%)	0.36
Peptoniphilus spp.	2 (1.7%)	0	2 (1.8%)	1
Clostridium spp.	1 (0.8%)	1 (10%)	0	1
Fusobacterium spp.	2 (1.7%)	0	2 (1.8%)	1
Urine culture positive	21 (17.5%)	8 (80%)	13 (12%)	<0.001
Blood culture positive	4 (3.3%)	4 (40%)	0	1

The overall median age was 84 years (range: 50-105), and the median BMI was 17.9 kg/m^2^, and 58% of patients were underweight, whereas 23% were severely underweight. No overweight patients were identified.

The median abscess size was 33 mm, although accurate pre-rupture measurements were unavailable in severe cases due to concurrent UR or rectovaginal fistula. Severe cases had significantly larger abscesses than non-severe cases (77.5 mm vs. 31.5 mm, p<0.01).

Most patients (83%) had an ECOG PS of three to four, indicating high dependency in daily activities. Interestingly, a higher proportion of severe cases had good pre-onset activities of daily living (ADL) measures, with PS zero to one (30% vs. 3.6%, p<0.05) and PS zero (20% vs. 1.8%, p<0.05) compared to non-severe ones. Residential status did not differ significantly between groups, with nursing homes being the most common setting.

Dementia was significantly less common in severe cases than non-severe ones (60% vs. 89%, p<0.05). Microbiologically, ESBL-producing *Escherichia coli* in vaginal pus and positive urine cultures were significantly more frequent in the severe group.

Case series of severe cases

Table 2 summarizes the clinical course of 10 severe cases.

**Table 2:** Characteristics of the 10 severe cases with pyometra uteri IV-Abx, intravenous-antimicrobial therapy; ATH, abdominal total hysterectomy; BMI, body mass index; BSO, bilateral salpingo-oophorectomy; CMZ, cefmetazole; ESBL, extended-spectrum β-lactamase; FMOX, flomoxef; TOA, tubo-ovarian abscess; UR, uterine rupture; VCM, vancomycin.

Case no.	Age	BMI	PS	Residential setting	Vaginal pus culture	Urine culture	Blood culture	Gynecologic complications	Treatment		Outcome
	(years)	(kg/m^2^)							IV-Abx	Surgical treatment	
1	88	16.7	3	nursing home	*Escherichia coli* (ESBL)	*Escherichia coli* (ESBL)	Anaerococcus prevotii	UR	FMOX	transvaginal drainage, repair of uterine defect	recovery
												2	73	20.3	3	nursing home	*Escherichia coli* (ESBL), *Enterococcus faecalis *	*Escherichia coli* (ESBL)	not detected	rectovaginal fistula, UR	CMZ	transvaginal drainage, ATH+BSO, Hartmann ope	recovery
3	92	19.5	4	nursing home	*Escherichia coli* (ESBL)	*Escherichia coli* (ESBL)	not detected	UR	CMZ	transvaginal drainage	death												
4	61	19.2	1	long-term care sanatorium	Escherichia coli	Escherichia coli	not detected	endometrial cancer, UR	FMOX	transvaginal drainage	death
												5	80	18.4	2	Independent living	Klebsiella pneumoniae	*Klebsiella pneumoniae*, *Streptococcus* spp.	Anaerococcus prevotii	UR	CMZ	ATH+BSO	recovery
6	50	21	0	Independent living	Escherichia coli	not detected	not detected	Adenomyosis, TOA, UR	FMOX	ATH+BSO	recovery												
7	82	17.3	4	nursing home	*Enterococcus faecalis*, *Corynebacterium *spp.	not detected	not detected	UR	CMZ	transvaginal drainage	death
												8	91	15.7	4	nursing home	*Escherichia coli*, *Clostridium* spp.	Escherichia coli	not detected	UR	VCM	ATH+BSO	death
9	71	19.8	0	Independent living	Bacteroides fragilis	Escherichia coli	*Bacteroides fragilis*, *Dialister* spp.	Adenomyosis, myoma, UR	VCM	ATH+BSO	death												
10	77	20.5	3	nursing home	*Escherichia coli* (ESBL), *Klebsiella pneumoniae*	*Escherichia coli* (ESBL)	*Bacteroides fragilis*, *Streptococcus* spp.	UR	VCM+CMZ	ATH+BSO	recovery

All were postmenopausal women presenting with UR. The median age was 78.5 years (IQR: 71.5-86.5), and the median BMI was 19.4 kg/m^2^ (IQR: 17.6-20.2). The in-hospital mortality rate among the severe cases was 50% (5/10), and overall mortality among all PU patients was 4.2%. All patients received broad-spectrum intravenous antibiotics. Three patients (cases three, four, and seven) underwent transvaginal drainage without laparotomy due to poor general condition; all three subsequently died. Six patients underwent abdominal total hysterectomy (ATH) and bilateral salpingo-oophorectomy (BSO). One patient (case one) was initially suspected of having a gastrointestinal perforation. During surgery, UR was identified, and the uterine defect was repaired without hysterectomy by a gastrointestinal surgeon. The patient recovered well. Another patient (case two) had UR and rectovaginal fistula due to infection, she underwent ATH and BSO, Hartmann’s operation, and repair of the rectovaginal fistula. In seven of the 10 cases, the microorganisms detected by vaginal pus culture were also detected by urine bacterial culture. Four patients had positive blood cultures, but only one of them died (case nine).

Logistic regression analysis for severe PU 

To explore factors associated with severe PU, univariate logistic regression analyses were performed (Table 3).

Because of the limited number of severe cases, multivariable logistic regression was not conducted. Several clinical and microbiological variables showed statistically significant associations with severe PU in univariate analyses. These included fever (OR=43.75, p<0.001), abdominal pain (OR=27.72, p<0.001), presence of coexisting adenomyosis (OR=16.55, p<0.05), detection of ESBL-producing *E. coli* in vaginal pus (OR=15.66, p<0.01), positive urine culture (OR=35.57, p<0.001), and larger abscess size (OR=1.04 per mm increase, p<0.01).

**Table 3:** Univariate logistic regression analysis for severe cases of pyometra uteri *p<0.05, **p<0.01; Adjusted for age and BMI. ADL, activities of daily living; BMI, body mass index; CI, confidence intervals; ESBL, extended-spectrum β-lactamase; OR, odds ratio; PS, performance status.

Variables	Unadjusted OR	95% CI		p-value	Adjusted OR		95% CI		p-value
Age	0.97	0.96	0.978	<0.001	-		-	-	-
BMI	1.068	0.87	1.32	0.54	-		-	-	-
Pre-onset ADL									
ADL (PS 4)	0.058	0.018	0.18	<0.001	0.52		0.123	2.18	0.37
ADL (PS 3)	0.071	0.02	0.23	<0.001	0.98		0.22	4.36	0.98
ADL (PS 2)	0.083	0.0073	0.701	0.38	0.91		0.01	8.11	0.93
ADL (PS 1)	6	0.49	72.72	0.16	3.25		0.21	51.2	0.4
ADL (PS 0)	13.5	1.67	108.86	0.015*	5.49		0.43	70.22	0.19
ADL (PS 0-1)	11.36	2.11	61	0.0046**	6.89		0.77	61.42	0.084
ADL (PS 0-2)	3.92	0.99	15.43	0.051	2.6		0.54	12.55	0.23
Residential settings									
Nursing home	0.76	0.202	2.86	0.69	1.05		0.26	4.28	0.95
Long-term care sanatorium	1	0.12	8.65	1	0.77		0.08	7.12	0.82
Home (independent living)	2.52	0.59	10.76	0.21	1.82		0.38	8.78	0.46
Symptoms									
Fever (38℃)	49.24	5.85	414.18	<0.001	43.75		5.12	373.79	<0.001
Abdominal pain	36	5.46	237.34	<0.001	27.72		3.85	199.81	<0.001
Atypical genital bleeding	0.85	0.17	4.26	0.84	0.67		0.13	3.53	0.63
Abnormal vaginal discharge	0.35	0.092	1.37	0.13	0.35		0.09	1.45	0.15
General comorbidities									
Hypertension	1.68	0.41	6.83	0.47	2.16		0.5	9.43	0.3
Hyperlipidemia	2.25	0.42	11.95	0.34	4.1		0.66	25.14	0.13
Cerebral vascular disease	0.155	0.019	1.26	0.081	0.21		0.02	1.78	0.15
Chronic heart disease	1.86	0.49	7.07	0.36	3.26		0.72	14.76	0.13
Chronic kidney disease	1.25	0.14	10.98	0.84	1.13		0.12	10.25	0.92
Osteoporosis	1	0.24	4.11	1	1.63		0.34	7.79	0.54
Dementia	0.29	0.075	1.146	0.077	0.51		0.1	2.48	0.4
OBGY comorbidities									
Endometrial cancer	12.11	0.69	210.2	0.087	3.61		0.14	95.53	0.44
Uterine cancer	6	0.49	72.72	0.16	2.55		0.14	48.12	0.53
Uterine myoma	2.25	0.42	11.95	0.34	1.49		0.23	9.65	0.68
Adenomyosis	27.25	2.22	333.81	0.0097**	16.55		1.005	272.41	0.049*
Culture of vaginal pus									
Escherichia coli	1.82	0.433	7.62	0.41	1.39		0.3	6.42	0.67
Escherichia coli (ESBL)	8.5	1.98	36.43	0.004**	15.66		2.86	85.75	0.0015**
Klebsiella pneumoniae	3.96	0.37	42.11	0.25	4.03		0.36	45.11	0.26
Enterococcus faecalis	12.11	0.69	210.2	0.086	18.2		0.95	349.7	0.054
Bacteroides spp.	2.94	0.29	29.21	0.36	2.94		0.27	32.25	0.38
Other test findings									
Urine culture positive	29.85	5.71	156.05	<0.001	35.57		5.91	214.25	<0.001
Abscess size (longest axis)	1.039	1.015	1.064	0.0011**	1.04		1.011	1.062	0.0041**

**Figure 1:**
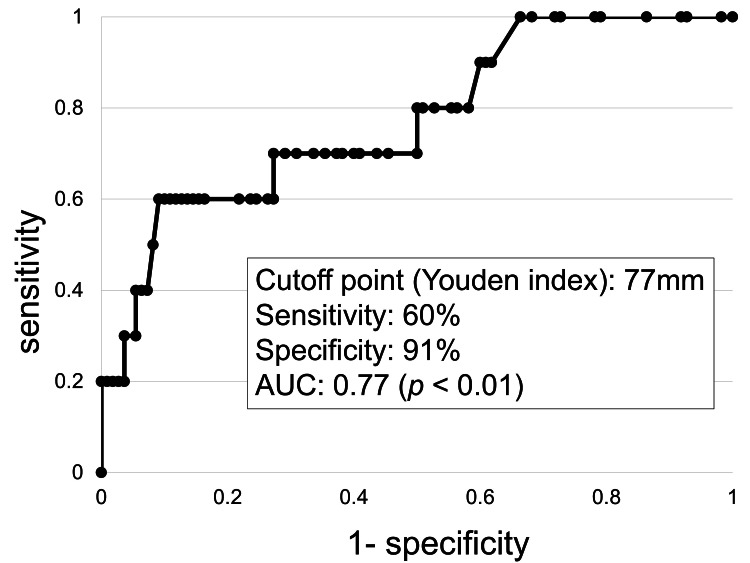
ROC curve of abscess size parameter for severe pyometra uteri AUC, area under the curve; PU, pyometra uteri; ROC, receiver operating characteristic.

ROC curve analysis for abscess size showed an optimal cut-off value of 77 mm, with a sensitivity of 60%, specificity of 91%, and an area under the curve or AUC of 0.77 (p<0.001; Figure 1). 

## Discussion

This retrospective study explored clinical and microbiological factors associated with severe PU in elderly patients treated at a single tertiary-care hospital. Although PU is usually managed conservatively, severe cases complicated by UR may result in life-threatening peritonitis and septic shock. In the present cohort, all severe cases involved UR, and the observed mortality rate was 50%, highlighting the potentially devastating consequences of this condition.

However, the results should be interpreted with caution. Only 10 severe cases were identified, which resulted in wide CIs in the regression analyses and limited statistical power. Consequently, the associations observed in this study should be regarded as preliminary and hypothesis-generating rather than definitive risk factors.

Abscess size

One of the notable findings was the association between larger intrauterine abscess size and severe disease. ROC analysis suggested a potential cutoff value of 77 mm for identifying severe cases. However, this threshold should be interpreted cautiously for several reasons.

First, the cutoff value was derived from the present dataset using the Youden index and has not been externally validated. Given the limited number of severe cases, there is a risk of overfitting. Second, the biological rationale for a specific threshold remains uncertain. While larger abscesses may increase intrauterine pressure and predispose the uterine wall to rupture, previous studies have not consistently proposed a clear size threshold associated with rupture.

Therefore, the 77-mm cutoff identified in this study should be considered exploratory. Future studies involving larger cohorts and independent datasets are necessary to determine whether a clinically meaningful threshold exists.

ESBL-producing *E. coli*

The detection of ESBL-producing *E. coli* in vaginal pus was another interesting observation. Previous studies of PU microbiology have reported common enteric organisms such as* E. coli*, *Klebsiella pneumoniae*, and anaerobic bacteria; however, multidrug-resistant strains have not been extensively discussed [8,12,13].

In the present study, ESBL-producing organisms were more frequently identified in severe cases. Nevertheless, the small number of isolates limits the strength of this observation. Furthermore, information regarding prior antibiotic exposure, healthcare-associated infection, or colonization with resistant organisms was not systematically available. These factors could potentially explain the observed association.

If confirmed in future studies, this finding could have implications for empirical antimicrobial therapy in severe or suspected complicated PU. At present, however, the result should be interpreted cautiously.

Urine culture findings

Another observation was the association between positive urine cultures and severe PU. This finding has rarely been reported previously. One possible explanation is a shared urogenital infection pathway, particularly in elderly women with postmenopausal mucosal changes, urinary incontinence, or lower urinary tract dysfunction [11,14].

However, the present study cannot establish a causal relationship between urinary tract infection and severe PU. The observed association may reflect increased bacterial burden, coexisting infection, or shared risk factors. Therefore, it is important to distinguish between statistical associations observed in the data and biological hypotheses proposed to explain them. Future studies could explore this hypothesis by comparing bacterial strains between urine and uterine cultures or by evaluating longitudinal microbiological data.

Surgical treatments

Regarding surgical treatment, there is no established standard for UR caused by PU due to the rarity of the condition. Most reported severe cases undergo ATH with BSO [2,3,6,13,15,16]. However, in the present study, one severe case (case one in Table 2) recovered fully with uterus-preserving surgery involving drainage and closure of the uterine defect, along with appropriate antimicrobial therapy. This minimally invasive approach aligns with recent case reports advocating for uterus-preserving surgery, especially in elderly or high-risk patients with septic shock [5,17-20]. Uterus preservation does not aim to retain fertility, but rather to minimize surgical invasiveness and support recovery in fragile patients. One notable case from the literature describes a staged surgical approach: initial uterine defect repair with peritoneal lavage and temporary abdominal closure, followed by planned ATH+BSO once the patient stabilized in the ICU [18]. This “damage control” strategy may offer significant survival advantages and should be considered, especially when initial surgery is performed by gastrointestinal surgeons unfamiliar with gynecologic procedures. Indeed, UR due to PU often mimics gastrointestinal perforation, and misdiagnosis is common [13,21]. This leads to gastrointestinal surgeons frequently performing emergency operations for these cases. For such surgeons, repairing the uterine defect - rather than performing a full hysterectomy - may be more feasible and safer in emergency settings. Collaboration between gynecologists and gastrointestinal surgeons is crucial for optimizing outcomes.

Case two (Table 2) in this study presented with a rectovaginal fistula caused by PU and required a multidisciplinary approach involving ATH+BSO and Hartmann’s procedure. This case underlines the importance of preoperative gastrointestinal evaluation in PU patients suspected of UR, as comorbid gastrointestinal diseases may coexist or complicate surgical decisions.

Study limitations

This study has several important limitations. First, the retrospective design and the small number of severe cases limit the statistical power and generalizability of the findings. Second, only limited adjustment for potential confounders was possible. Third, imaging protocols and measurement methods for abscess size were not standardized because imaging was performed as part of routine clinical care. Because of these limitations, the results should primarily be interpreted as exploratory findings that may inform future research rather than definitive evidence of causal relationships.

## Conclusions

In this retrospective cohort of elderly patients with PU, several clinical and microbiological variables, including systemic symptoms, larger abscess size, presence of coexisting adenomyosis, ESBL-producing *E. coli*, and positive urine cultures, were statistically associated with severe disease complicated by uterine rupture. However, because only a small number of severe cases were available for analysis, these findings should be interpreted cautiously. Rather than establishing definitive risk factors, the present results highlight potential variables that merit further investigation. Future multicenter prospective studies with larger cohorts and standardized diagnostic protocols are needed to validate these observations and clarify their implications for early risk stratification and clinical management of PU.
